# The evolution of the Global Burden of Disease framework for disease, injury and risk factor quantification: developing the evidence base for national, regional and global public health action

**DOI:** 10.1186/1744-8603-1-5

**Published:** 2005-04-22

**Authors:** Alan D Lopez

**Affiliations:** 1 School of Population Health, The University of Queensland, Brisbane, Australia

## Abstract

Reliable, comparable information about the main causes of disease and injury in populations, and how these are changing, is a critical input for debates about priorities in the health sector. Traditional sources of information about the descriptive epidemiology of diseases, injuries and risk factors are generally incomplete, fragmented and of uncertain reliability and comparability. Lack of a standardized measurement framework to permit comparisons across diseases and injuries, as well as risk factors, and failure to systematically evaluate data quality have impeded comparative analyses of the true public health importance of various conditions and risk factors. As a consequence the impact of major conditions and hazards on population health has been poorly appreciated, often leading to a lack of public health investment. Global disease and risk factor quantification improved dramatically in the early 1990s with the completion of the first Global Burden of Disease Study. For the first time, the comparative importance of over 100 diseases and injuries, and ten major risk factors, for global and regional health status could be assessed using a common metric (Disability-Adjusted Life Years) which simultaneously accounted for both premature mortality and the prevalence, duration and severity of the non-fatal consequences of disease and injury. As a consequence, mental health conditions and injuries, for which non-fatal outcomes are of particular significance, were identified as being among the leading causes of disease/injury burden worldwide, with clear implications for policy, particularly prevention. A major achievement of the Study was the complete global descriptive epidemiology, including incidence, prevalence and mortality, by age, sex and Region, of over 100 diseases and injuries. National applications, further methodological research and an increase in data availability have led to improved national, regional and global estimates for 2000, but substantial uncertainty around the disease burden caused by major conditions, including, HIV, remains. The rapid implementation of cost-effective data collection systems in developing countries is a key priority if global public policy to promote health is to be more effectively informed.

## Introduction

Whether it is through scientific curiosity, administrative edict or public health planning necessity, most countries have initiated some form of data collection and health surveillance/monitoring systems to provide information on health priorities. In some cases, such as the Bills of Mortality of the London Parishes, these attempts date back well over 300 years [1]. Cause of death statistics for the population of England and Wales have been collected for almost 200 years, and in most developed countries, for at least a century [2]. Further, many developed countries have instituted incidence registers for major diseases of public health importance, such as cancer, or routinely conduct health surveys to measure the prevalence of disease or risk factor exposures [3,4]. In poorer countries, national registration and certification of all deaths is less common, due to the cost of establishing and maintaining such a system, and often the mortality data collected are incomplete and of poor quality [5]. 'Verbal autopsy' procedures, using structured interviews with the family of the deceased, provide a history of symptoms experienced by the deceased, but translating these into reliable cause of death information for populations has only met with limited success [6-9]. Moreover, reliable information on the incidence and prevalence of diseases, injuries and risk factors is rarely available in developing countries, and what data are collected, particularly hospital records, are unlikely to reflect the true pattern of disease and injury in the community due to biases arising from the nature of conditions typically treated in hospitals and the ability of sectors of the population to afford tertiary care.

As a result, while most countries have some information about prevalence, incidence and mortality from some diseases and injuries, and some information on population exposure to risk factors, it is generally fragmented, partial, incomparable and diagnostically uncertain. Setting health priorities, however, requires, or at least should, information that is comparable, reliable and comprehensive across a wide range of conditions and exposures that cause death or ill-health in a population. The importance of capturing disease burden from largely non-fatal, but prevalent conditions such as depression or musculoskeletal conditions is critical. Substantial resources are usually invested by society to reduce their impact in populations, yet they rank extremely low among causes of mortality, the traditional basis upon which health priorities have been considered.

This paper describes a framework (the *Global Burden of Disease Study *[10]) for integrating, validating, analysing and disseminating fragmentary information on the health of populations so that it is truly useful for health policy and planning. Features of this framework include the incorporation of data on non-fatal health outcomes into summary measures of population health, the development of methods and approaches to estimate missing data and to assess the reliability of data, and the use of a common metric to summarise disease burden from both diagnostic categories of the International Classification of Disease and Injuries, and the major risk factors that cause those health outcomes. The approach has been widely adopted by countries and health development agencies alike as the standard for health accounting, as well as guiding the determination of health research priorities [11-14].

## Global Burden of Disease 1990 Study

The *Global Burden of Disease *(GBD) Study was commissioned by The World Bank in the early 1990s to provide a comprehensive assessment of disease burden in 1990 from over 100 diseases and injuries, and from 10 selected risk factors, for the world and 8 major World Bank regions [15-17]. The estimates were combined with research into the cost-effectiveness of intervention choices in different populations to develop recommended intervention packages for countries at different stages of development [18]. The methods and findings of the original (1990) GBD Study have been widely published [18-25], and have spawned numerous national disease burden exercises. The basic philosophy guiding the burden of disease approach is that there is likely to be information content in almost all sources of health data, provided they are carefully screened for plausibility and completeness; and that internally consistent estimates of the global descriptive epidemiology of major conditions are possible with appropriate tools, investigator commitment and expert opinion. To prepare estimates of the incidence, prevalence, duration and mortality from over 500 sequelae of more than 100 disease or injuries, a mathematical model, DISMOD, was developed for the 1990 GBD Study to convert partial, often non-specific data on disease/injury occurrence into a consistent age description of the basic epidemiological parameters in each Region [26].

To assess disease burden, a time-based metric which measured both premature mortality (years of life lost, or YLLs) and disability (years of life lived with a disability, weighted by the severity of the disability, or YLDs) was used. The sum of the two components, namely Disability-Adjusted Life Years, or DALYs, provides a measure of the future stream of healthy life (i.e. years expected to be lived in full health) lost as a result of the incidence of specific diseases and injuries in 1990. The effect of incident fatal cases (of disease or injury) is captured by YLLs, while the future health consequences, in terms of sequelae of diseases or injuries, of incident cases in 1990 that were not fatal, are measured by YLDs. A more complete account of the index, and the philosophy underlying parameter choices, is described elsewhere [27,28]. DALYs are not unique to the *Global Burden of Disease Study*. A variant of DALYs was used by The World Bank in the seminal Health Sector Priorities Review study [29], and derive more generally from earlier work to develop time-based measures that better reflect the public health impact of death or illness at younger ages [30,31]. DALYs are a particular (inverse) form of the more general concept of "Quality-Adjusted Life Years" or QALYs, proposed by Zeckhauser and Shepard in 1976 [32] and widely used in economic evaluations. Much of the comment and criticism of the GBD Study has focussed on the construction of DALYs [33-35], particularly the social choices around age-weights and severity scores for disabilities, and relatively little around the vast uncertainty of the basic descriptive epidemiology, especially in Africa, which is likely to be far more consequential for setting health priorities [36].

The results of the study confirmed what many health workers in mental health promotion and injury prevention had suspected for some time, namely that neuropsychiatric disorders on the one hand, and injuries on the other, were major causes of lost years of healthy life, as measured by DALYs. Table 1 summarises the major causes of disease burden worldwide in 1990 from among the 100 or so specific conditions quantified in the Study. The Table also lists the leading causes of premature mortality, as well as disability, as measured by YLLs and YLDs, respectively. Globally, in 1990, the leading causes of childhood diseases (lower respiratory diseases, diarrhoeal diseases, and perinatal causes such as birth asphyxia, birth traumas and low birth weight) were also the leading causes of disease burden, in part because of their concentration at younger ages. Interestingly, depression ranked fourth, ahead of ischaemic heart disease, cerebrovascular disease, tuberculosis and measles. Road traffic accidents also ranked in the top 10 causes of DALYs worldwide. Using more broad disease categories, non-communicable diseases, including neuropsychiatric disorders, were estimated to have caused 41% of the global burden of disease in 1990, only slightly less than communicable, maternal, perinatal and nutritional conditions combined (44%), with 15% due to injuries [10]. The class of infectious and parasitic diseases were the cause of more than one in five (23%) DALYs lost in 1990, followed by neuropsychiatric conditions (10.5%), cardiovascular diseases (9.7%), respiratory infections (8.5%), perinatal conditions (6.7%) and cancers (5.1%).

**Table 1:** Leading causes of premature mortality, disability and disease burden, World, 1990

Premature Mortality				Disability			Disease Burden		
	
									
**Rank**	**Disease/ injury**	**YLLs (000s)**	**Cumulative %**	**Disease/injury**	**YLDs (000s)**	**Cumulative%**	**Disease/injury**	**DALYs (000s)**	**% of Total**

1	Lower res. inf.	108601	12.0	Depression	50810	10.7	Lower res. inf.	112898	8.2
2	Diarrhoeal dis.	94434	22.4	Iron def. anaem.	21987	15.4	Diarrhoeal dis.	99633	7.2
3	Perinatal cond.	82681	31.5	Falls	21949	20.0	Perinatal cond.	92313	6.7
4	Isch. heart dis.	41595	36.1	Alcohol use	15770	23.4	Depression	50810	3.7
5	Measles	36450	40.1	COPD^1^	14692	26.5	Isch. heart dis.	46699	3.4
6	Tuberculosis	34304	43.9	Bipolar dis.	14141	29.5	Cerebrovas. dis.	38523	2.8
7	Cerebrovas. Dis.	32115	47.5	Congenital anom	13507	32.3	Tuberculosis	38426	2.8
8	Malaria	28038	50.5	Osteoarthritis	13275	35.1	Measles	36520	2.7
9	Road traffic acc.	26162	53.4	Schizophrenia	12183	37.7	Road traffic acc.	34317	2.5
10	Congenital anom.	19414	55.6	Obs.-comp dis^2^	10213	39.9	Congenital anom.	32921	2.4

*Source *Murray and Lopez (10)

^1 ^Chronic obstructive pulmonary disease

^2 ^Obsessive-compulsive disorders

By and large, the leading causes of years of potential life lost (YLLs) were similar, the major difference being that depression is not a major cause of premature mortality. It is, however, a major cause of non-fatal disease burden, causing more than 10% of all years lived with a disability (YLDs) worldwide, more than twice the contribution from the next leading cause, anaemia (4.7%). Indeed, as Table 1 shows, five of the top 10 leading causes of disability in 1990, as measured by YLDs, were neuropsychiatric conditions.

For prevention, comparative estimates of the disease and injury burden caused by exposure to major risk factors is likely to be a much more useful guide to policy action and priorities than a 'league table' of disease and injury burden alone. Over the past few decades, epidemiologists have attempted to quantify the impact of specific exposures, particularly tobacco, on mortality, either from major diseases such as cancer [37,38], or across a group of countries using comparable methods [39,40]. Specific country studies have examined the impact of several leading risk factors [41,42], but prior to the GBD Study, there was no global assessment of the fatal and non-fatal disease and injury burden from exposure to major health hazards. Ten such hazards (see Table 2) were quantified in the 1990 Study, based on information about causation, prevalence, exposure, and disease and injury outcomes available at the time. Almost one-sixth of the entire global burden of disease and injury that occurred in 1990 was attributed to malnutrition, another 7% or so to poor water and sanitation, and 2–3% from risks such as unsafe sex, tobacco, alcohol and occupational exposures.

**Table 2:** Global burden of disease and injury attributable to selected risk factors, 1990

**Risk factor**	**Deaths (thousands)**	**As % of total deaths**	**YLLs (thousands)**	**As % of total YLLs**	**YLDs (thousands)**	**As % of total YLDs**	**DALYs (thousands)**	**As % of total DALYs**
Malnutrition	5 881	11.7	199 486	22.0	20 089	4.2	219 575	15.9
Poor water supply sanitation and personal and domestic hygiene	2 668	5.3	85 520	9.4	7 872	1.7	93 392	6.8
Unsafe sex	1 095	2.2	27 602	3.0	21 100	4.5	48 702	3.5
Tobacco	3 038	6.0	26 217	2.9	9 965	2.1	36 182	2.6
Alcohol	774	1.5	19 287	2.1	28 400	6.0	47 687	3.5
Occupation	1 129	2.2	22 493	2.5	15 394	3.3	37 887	2.7
Hypertension	2 918	5.8	18 665	1.9	1 411	0.3	19 076	1.4
Physical inactivity	1 991	3.9	11 353	1.3	2 300	0.5	13 653	1.0
Illicit drugs	100	0.2	2 634	0.3	5 834	1.2	8 467	0.6
Air pollution	568	1.1	5 625	0.6	1 630	0.3	7 254	0.5

*Source: *Murray and Lopez (10)

## Improving Comparative Quantification of Diseases, Injuries and Risk Factors: The Global Burden of Disease 2000 Study

The initial *Global Burden of Disease Study *represented a quantum leap in the global and regional quantification of the impact of diseases, injuries and risk factors on population health. The results of the study have been widely used by government and non-governmental agencies alike to argue for more strategic allocation of health resources to disease prevention and control programs that are likely to yield the greatest gains in population health. Following the publication of the initial study, several national applications of the methods have led to substantially more data on the descriptive epidemiology of diseases and injuries, as well as to improvements in analytical methods. Critiques of the approach, and particularly of the methods used to assess the severity weightings for disabling health states, have led to fundamental changes in the way that health state valuations are determined (population-based rather than expert opinion as used in the 1990 study), and to substantially better methods for improving the cross-national comparability of survey data on health status [43,44]. Better methods for modelling the relationship between the level of mortality and the broad cause structure in populations, based on proportions rather than rates, have led to greater confidence in cause of death estimates for developing countries [45]. Improved population surveillance for some major diseases such as HIV/AIDS, and the wider availability of data from 'verbal autopsy' methods, particularly in Africa, has lessened the dependence on models for cause of death estimates, although substantial uncertainty still remains in the use of such data.

Perhaps the major methodological progress since the GBD 1990 Study has been with respect to risk-factor quantification. In the initial study, the population health effects of 10 risk factors were quantified, but there are serious concerns about the comparability of the estimates. Different risk factors have very different epidemiological traditions, particularly with regard to the definition of "hazardous" exposure, the strength of evidence on causality, and the availability of epidemiological research on exposure and outcomes. As a result, comparability across estimates of disease burden due to different risk factors is difficult to establish. Moreover, classical risk factor research has treated exposures as dichotomous, with individuals either exposed or non-exposed, with exposure defined according to some, often arbitrary, threshold value. Recent evidence for such continuous exposures as cholesterol, blood pressure and body mass index suggests that such arbitrarily defined thresholds are inappropriate, since hazard functions for these risks decline continuously across the entire range of measured exposure levels, with no obvious threshold [46,47] For the GBD 2000 Study, a new framework for risk factor quantification was defined which, instead of the classical dichotomous approach, measured changes in disease burden that would be expected under different population distributions of exposure [48] Attributable fractions of disease due to a risk factor were then calculated based on a comparison of disease burden expected under the current (i.e. 2000) estimated distribution of exposure, by age, sex and Region, with that expected if a *counterfactual *distribution of exposure had applied. The counterfactual distribution was defined for each risk factor as the population distribution of exposure that would lead to the lowest theoretical minimum levels of disease burden. Thus, for example, in the case of tobacco, the theoretical minimum distribution would be 100% of the population being life-long non-smokers; for BMI it would be 100% of the population having a BMI of 21 (SD1) kg/m^2^, and so on. The theoretical minima for each of the risk factors quantified in the WHO *Comparative Risk Assessment (CRA) *study (the risk factor arm of the GBD 2000 Study) were developed by expert groups for each risk factor and are described in more detail elsewhere [49,50].

The main findings of the CRA Study are summarized in Table 3. In all, 26 risk factors were quantified, each by age and sex, and within 14 WHO epidemiological Regions, as well as for the world. These regions were further grouped into "developed" "low-mortality developing" including China and much of Latin America, and "high mortality developing" including Sub-Saharan Africa, and many countries in Western and Southern Asia, including India, Bangladesh and Myanmar. As the table suggests, the world is currently experiencing a "risk factor" transition, with developed countries characterized by high disease burden from tobacco, sub-optimal blood pressure, alcohol, cholesterol and overweight. Disease burden in the poorest countries, on the other hand, is primarily caused by underweight, unsafe sex, unsafe water and sanitation, indoor air pollution and micronutrient deficiencies (zinc, iron, vitamin A). Interestingly, the risk factors which, on average, cause the greatest disease burden among the 2.4 billion people living in low-mortality developing countries are a mixture of both, led by alcohol, sub-optimal blood pressure and tobacco, followed by underweight and overweight. This juxtaposition of what might be termed "new" and "old" risk factors strongly suggests that health policy in developing countries must increasingly address risks such as tobacco and blood pressure that have often mistakenly been labelled, and treated, as conditions of affluence.

**Table 3:** Leading risk factors for disease burden in 2000, by development category

**Developing countries**		**Developed countries**	
**High mortality countries**	**% of Total DALYs**		**% of Total DALYs**

Underweight	14.9%	Tobacco	12.2%
Unsafe sex	10.2%	Blood pressure	10.9%
Unsafe water, sanitation and hygiene	5.5%	Alcohol	9.2%
Indoor smoke from solid fuels	3.6%	Cholesterol	7.6%
Zinc deficienty	3.2%	Overweight	7.4%
Iron deficiency	3.1%	Low fruit and vegetable intake	3.9%
Vitamin A deficiency	3.0%	Physical inactivity	3.3%
Blood pressure	2.5%	Illicit drugs	1.8%
Tobacco	2.0%	Unsafe sex	0.8%
Cholesterol	1.9%	Iron deficiency	0.7%

**Low mortality countries**	**% of Total DALYs**		

Alcohol	6.2%		
Blood pressure	5.0%		
Tobacco	4.0%		
Underweight	3.1%		
Overweight	2.7%		
Cholesterol	2.1%		
Low fruit and vegetable intake	1.9%		
Indoor smoke from solid fuels	1.9%		
Iron deficiency	1,8%		
Unsafe water, sanitation and hygiene	1.8%		

*Source*: World Health Organization (46)

## Improving Cross-Population Comparability of Disease Burden Assessments

While the first *Global Burden of Disease Study *set new standards for measuring population health, the basic units of analysis for the study were the 8 World Bank Regions defined for the 1993 *World Development Report*. Designed to be geographically contiguous, these Regions were nonetheless extremely heterogenous with respect to health development. Other Asia and Islands (OAI) for example, included countries with such diverse epidemiological profiles as Singapore and Myanmar. This seriously limits their value for comparative epidemiological assessments. For the *Global Burden of Disease 2000 Study*, a more refined approach was followed. Estimates of disease and injury burden were first developed for each individual Member State of WHO (191 in 2000) using different methods for countries at different stages of health development, often largely determined by the availability of data [51]. For example, age-sex-specific death rates for countries were essentially determined using one of three standard approaches: routine life-table methods for countries with complete vital registration; application of standard demographic methods to correct for under-registration of deaths; or, where no vital registration data on adult mortality were available, application of model life tables [51,52].

The detailed methodological approaches adopted for countries to estimate cause-specific mortality, and the descriptive epidemiology of non-fatal conditions in each country are described elsewhere [53]. This focus on individual countries as the unit of analysis, as well as the systematic application of standardized approaches for all countries in any given category of data availability, has vastly improved the cross- population comparability of disease and injury quantification, at least among countries at similar levels of health development.

Caution is required, however, in inferring comparability of national disease burden assessments across countries at different levels of development. Estimates of mortality in countries where there is no functioning vital registration system for causes of death will always be substantially more uncertain than those derived from systems where all deaths are registered and medically certified, as is the case for developed countries. For example, in the United States, uncertainty around the mean life expectancy for males in 2000 (73.9 years) was ± 0.3 years, compared to ± 3.5 years in Uganda [51]. The same may be said for the quantification of disability due to various conditions, where the gap in data availability between rich and poor countries is likely to be even more extreme than for mortality. A major advance with the *Global Burden of Disease 2000 Study *has been the systematic attempt to quantify uncertainty in both national and global assessments of disease burden. This uncertainty must be taken into account when making cross-national comparisons, and needs to be carefully communicated and interpreted by epidemiologists and policy makers alike.

To date, systematic national estimates of the burden of disease due to major risk factors, applying the standardized framework of the *Comparative Risk Assessment Project*, have not been attempted. Standardized approaches to measuring mortality attributable to some risk factors, such as tobacco, have been developed and applied to 50 or so developed countries [39], but more research is urgently needed to prepare comparative risk estimates, by country, using the broader, more comprehensive CRA framework. There is no *a priori *reason to expect that the uncertainties in cross-national comparisons for risk factors would be any greater than those for diseases and injuries that have already been quantified.

## Discussion and Conclusions

*The World Development Report 1993 *provided an enormous impetus to the development of global and regional quantification of disease and injury burden, and of what causes it. The vast exercise in global descriptive epidemiology that was required to develop estimates led to the first ever comprehensive estimates of the fatal and non-fatal burden for over 100 diseases and injuries, as well as for selected risk factors. The development and widespread application of a single summary measure of population health (DALYs) has greatly facilitated scientific and political assessments of the comparative importance of various diseases, injuries and risk factors, particularly for priority-setting in the health sector, and has led to strategic decisions by some agencies eg. WHO, to invest greater effort in program developments to address priority health concerns such as tobacco control and injury prevention. The subsequent *Global Burden of Disease 2000 Study*, and a plethora of country applications, have led to substantial improvements in both methods and data availability, as well as in the comparability of results. They have not, however, led to significant changes in the comparative magnitude of most conditions, the single exception being HIV/AIDs, largely as a result of the explosion of the epidemic during the 1990s in Southern Africa. Nor have these methodological advances adequately addressed the challenges that arise from new data sets becoming available. For example, better methods are needed to estimate adult mortality levels from survey data [54], to estimate biases in using hospital data to infer community-level cause of death patterns, and to more reliably quantify the joint effects of multiple risks acting in concert to produce disease outcomes.

This relative stability in the outcomes of disease and risk factor quantification does not necessarily inspire greater confidence that the estimates are correct. Rather, it suggests that despite the progress of the past decade, the incremental gains in advancing our knowledge and understanding of global descriptive epidemiology have been modest. There is an urgent need for a globally-coordinated research and development effort to devise and implement cost-effective approaches to data collection and analysis in poor countries that is targeted to their health development needs, and that can routinely yield comparable information of sufficient quality to establish how disease and risk factor burden is changing in populations. Recent calls for the establishment of a global health monitoring Centre to continuously assess, using comparable methods, the impact of diseases, injuries and risk factors worldwide are a step in this direction [55], but much more needs to be done to assist countries with the development of minimal health information systems. It is lamentable how little is reliably known about the global impact of diseases, injuries and risk factors. It would be unconscionable if we were to be similarly ignorant 10 to 20 years hence.
